# *Journal of Hematology & Oncology* reviewer acknowledgement 2015

**DOI:** 10.1186/s13045-016-0245-6

**Published:** 2016-03-02

**Authors:** Delong Liu

**Affiliations:** Department of Medicine, New York Medical College and Westchester Medical Center, New York, NY USA

## Abstract

The Editor-in-Chief of *Journal of Hematology & Oncology* would like to thank all our reviewers who have contributed to the journal in Volume 8 (2015).

**Nacer Abrouk**

United States of America

**Akintunde Akinleye**

United States of America

**Yoshimitsu Akiyama**

Japan

**Ugo Ala**

Italy

**A Alousi**

United States of America

**Xiuli An**

United States of America

**Ephraim Ansa-Addo**

United States of America

**Emmanuel Antonarakis**

United States of America

**Dorina Avram**

United States of America

**Veronika Bachanova**

United States of America

**Veronica Balatti**

United States of America

**Srinivasa Rao Bandi**

United States of America

**Frederic Baron**

Belgium

**Rodolphe Barrangou**

United States of America

**Giuseppe Basso**

Italy

**Ioana Berindan-Neagoe**

Romania

**Paolo Bernasconi**

Kazakhstan

**Jin-Song Bian**

Singapore

**Andrea Biondi**

Italy

**Cf Boerkoel**

Canada

**Arnold Bolomsky**

Austria

**Earle Burgess**

United States of America

**Hong Chang**

Canada

**Ying-Jun Chang**

China

**Huan Chen**

China

**Suning Chen**

China

**Guoan Chen**

United States of America

**M Chen**

United States of America

**Vicky Ping Chen**

United States of America

**Tao Cheng**

China

**Hong Cheng**

United States of America

**Seok-Goo Cho**

South Korea

**Eun Young Choi**

Korea, South

**Shih-Sung Chuang**

Taiwan

**Mei-Zhen Cui**

United States of America

**Silvia Deaglio**

Italy

**Jiusheng Deng**

United States of America

**Fei Duan**

United States of America

**William Durante**

United States of America

**Xiucheng Fan**

United States of America

**Qipeng Fan**

United States of America

**Zhichao Fan**

United States of America

**Mark Feinberg**

United States of America

**Michael Flister**

United States of America

**Karlheinz Friedrich**

Germany

**Yanfang Fu**

United States of America

**Seiji Fukuda**

Japan

**Claudia Fumarola**

Italy

**Sara Galimberti**

Italy

**S Gallego**

Spain

**Gregory Gan**

United States of America

**Joan Gil**

Spain

**Alois Gratwohl**

United States of America

**Charlotte Grootaert**

Belgium

**Aihua Gu**

China

**Ming Guan**

China

**Christina Halsey**

United Kingdom

**Lina Han**

United States of America

**Bo Han**

United States of America

**Donald Harvey**

United States of America

**Lizhi He**

United States of America

**Yukai He**

United States of America

**Mark Heaney**

United States of America

**Cyrus Hedvat**

United States of America

**Tom Hobman**

Canada

**Jian Hou**

China

**George Hsiao**

Taiwan

**Wenming Hsu**

United States of America

**Chung-Tsen Hsueh**

United States of America

**Qinglong Hu**

United States of America

**Shimin Hu**

United States of America

**Wenwei Hu**

United States of America

**He Huang**

China

**Xiaojun Huang**

China

**Huang Huang**

United States of America

**Suming Huang**

United States of America

**Tiangui Huang**

United States of America

**Tsong-Long Hwang**

Taiwan

**William Hwang**

Singapore

**Toshihiko Imamura**

Japan

**Benjamin Jackson**

United States of America

**Gaetan Jego**

France

**Yuxia Jia**

United States of America

**Wei Jiang**

United States of America

**Dan Jones**

United States of America

**Celina Juliano**

United States of America

**Hirokazu Kanegane**

Japan

**Khosrow Kashfi**

United States of America

**Xiao Yan Ke**

China

**Fadi Khasawneh**

United States of America

**Joseph Khoury**

United States of America

**Edward Kim**

United States of America

**Seok Jin Kim**

South Korea

**Won Seog Kim**

South Korea

**M Kitagawa**

Japan

**Maria Kleppe**

United States of America

**Ilona Kovalszky**

Hungary

**Dieter Kube**

Germany

**Y Lai**

China

**Gurpreet Lamba**

United States of America

**Jungwoo Lee**

United States of America

**Mong-Hong Lee**

United States of America

**Robert Lerner**

United States of America

**Chi-Kwong Li**

Hong Kong

**Jin Li**

China

**Ji-Liang Li**

United Kingdom

**Peng Li**

China

**Yong Li**

United States of America

**Qiao Li**

China

**Shaoguang Li**

United States of America

**Tianhong Li**

China

**Guangfu Li**

United States of America

**Shaoguang Li**

United States of America

**Yangqiu Li**

China

**Zhenyu Li**

United States of America

**Zihai Li**

United States of America

**Felix Lichtenegger**

Germany

**Edward Lin**

United States of America

**Jianqing Lin**

United States of America

**Hongtao Liu**

United States of America

**Jianguo Liu**

United States of America

**Qi-Fa Liu**

China

**Zach Liu**

United States of America

**Sanam Loghavi**

United States of America

**Min Lu**

China

**Harry Lybeck**

United States of America

**Manisha Madkaikar**

United States of America

**Atsushi Manabe**

Japan

**Luigi Marcheselli**

Italy

**Philip Mason**

United States of America

**Rohit Mathur**

United States of America

**Sebastien Maury**

France

**Helmout Modjtahedi**

United Kingdom

**S Montes-Moreno**

Spain

**Hideki Muramatsu**

Japan

**Hiroshi Nakano**

Japan

**Heyu Ni**

Canada

**Maria Odero**

Spain

**Seiichi Okabe**

Japan

**Dennis O'malley**

United States of America

**Thomas Pabst**

Switzerland

**Minggui Pan**

United States of America

**Gyorgy Panyi**

Hungary

**Simrit Parmar**

United States of America

**Luiz O. Penalva**

United States of America

**Piergiorgio Petroni**

Italy

**Simon Powis**

United Kingdom

**B Psaila**

United Kingdom

**Zhenggang Ren**

China

**Ruibao Ren**

United States of America

**Uwe Reusch**

Germany

**Jia Ruan**

United States of America

**Ko Sato**

Japan

**Martin Sauvageau**

United States of America

**Tobias Schatton**

United States of America

**Ig Schmidt-Wolf**

Germany

**Karen Seiter**

United States of America

**Lohith Shakaladevanapura**

United States of America

**He Shen**

United States of America

**Yuankai Shi**

China

**Sy Shieh**

United States of America

**Yongqian Shu**

China

**Irma Rosa Slavutsky**

Argentina

**Jürgen Sonnemann**

Germany

**Carmen Stanganelli**

Argentina

**Gp Studzinski**

United States of America

**Vivek Subbiah**

United States of America

**Marion Subklewe**

Germany

**M Suenaga**

Japan

**Hui-Chuan Sun**

China

**Daniel Sze**

Australia

**Andrew Tan**

Singapore

**Guilin Tang**

United States of America

**Jianguo Tao**

United States of America

**Jessica Thaxton**

United States of America

**Erming Tian**

United States of America

**Raoul Tibes**

United States of America

**Ramon Tiu**

United States of America

**K Tokoyoda**

Germany

**Che-Kai Tsao**

United States of America

**Panagiotis Tsirigotis**

Israel

**Jeffrey Tyner**

United States of America

**Ikuyo Ueda**

Japan

**Diamantina Vasilatou**

Greece

**Amit Verma**

United States of America

**Massimiliano Visocchi**

United States of America

**Phuong Vo**

United States of America

**Sinisa Volarevic**

Croatia

**Jing Wang**

United States of America

**Jiyi Wang**

United States of America

**Sa Wang**

United States of America

**Qinhong Wang**

United States of America

**Wei Wang**

United States of America

**Xiaoxiao Wang**

United States of America

**Britta Will**

United States of America

**Petter Woll**

United States of America

**Justin Wong**

Australia

**John Wrangle**

United States of America

**Kongming Wu**

China

**Shenhong Wu**

United States of America

**Yi-Long Wu**

China

**Jingjing Wu**

China

**Lijun Xia**

United States of America

**Zhijian Xiao**

China

**Yu-Quan Xiong**

China

**Binghe Xu**

China

**Dazhong Xu**

United States of America

**Ruihua Xu**

China

**Ozlem Yalcin**

United States of America

**Masayuki Yamamoto**

Japan

**Shuo Yang**

United States of America

**Naohiro Yano**

United States of America

**Jeffrey Ye**

United States of America

**Go Yoshida**

Japan

**Scott Younger**

United States of America

**Evan Yu**

United States of America

**Min Yu**

United States of America

**Jianda Yuan**

United States of America

**Zihua Zeng**

United States of America

**Hua Zhang**

China

**Lanjing Zhang**

United States of America

**Chengcheng Zhang**

United States of America

**Xi Zhang**

United States of America

**Xu Zhang**

China

**Yue Zhang**

United States of America

**Jing Zhang**

United States of America

**Ti Zhang**

China

**Wei-Li Zhao**

China

**Guo-Guang Zheng**

China

**Xiaoyong Zheng**

United States of America

**Huyong Zheng**

China

**Wenxin Zheng**

United States of America

**Xiaoyong Zheng**

United States of America

**Yong Zhou**

China

**Niklas Zojer**

Austria

